# Strong ERG Positivity in Papillary Intralymphatic Angioendothelioma of the Testis of a 24-Year-Old Male: A Case Report

**DOI:** 10.1155/2013/531479

**Published:** 2013-03-31

**Authors:** Anne M. Schultheis, Mareike Sandmann, Stefan Steurer

**Affiliations:** ^1^Institute of Pathology, University of Cologne Medical Center, 50937 Cologne, Germany; ^2^Institute of Pathology, University Hospital Hamburg Eppendorf, 20246 Hamburg, Germany

## Abstract

Papillary intralymphatic angioendothelioma is a rare, low-grade neoplasm of lymphatic channels that usually presents intradermally or subcutaneously. We report the case of a 24-year-old male presenting with an isolated intratesticular palpable mass and symptoms of testicular pain. Preoperative ultrasound examination showed an irregular, heterogeneous mass. Subsequent surgery and pathologic assessment revealed a papillary intralymphatic angioendothelioma (PILA), formerly known as Dabska tumor within the lymphatic spaces.

## 1. Introduction

Papillary intralymphatic angioendothelioma, formerly known as Dabska tumor, was first described in 1969 by Dabska [1]. The term Dabska tumor has essentially fallen into disuse, since the lesions described herein included other tumors that would nowadays be diagnosed as retiform hemangioendothelioma. It is a rare vascular neoplasia characterized by endovascular papillary proliferations of atypical endothelial cells and anastomosing vascular channels. Some of these contain papillary projections or tuft-like structures, resembling renal glomeruli. It mostly affects the skin or the subcutaneous tissue of children. Only a few cases describe its occurrence in deeper tissue, and so far, only one other case of testicular involvement has been described in the literature [2–5].

## 2. Case Report

A 24-year-old male initially consulted his urologist for pain and tenderness in the scrotum. There was no previous history of genital trauma. On examination, the scrotum showed a discrete swelling of the upper pole of the left testis. There was no abnormality to the right testis. Ultrasound examination revealed a circumscribed mass with heterogenous echotexture. The possibility of a testicular tumor was suggested, and the patient was sent to hospital for further investigation. Subsequently, orchidectomy was carried out. The specimen was sent for histopathological examination.

The gross examination revealed a tumor located centrally in the testicular parenchyma; the shape of the testis was maintained. The tumor measured 1.4 cm in diameter. Outer surface was smooth and brownish in colour. The cut surface was reddish brown with areas of hemorrhage. 

**Figure 1:**
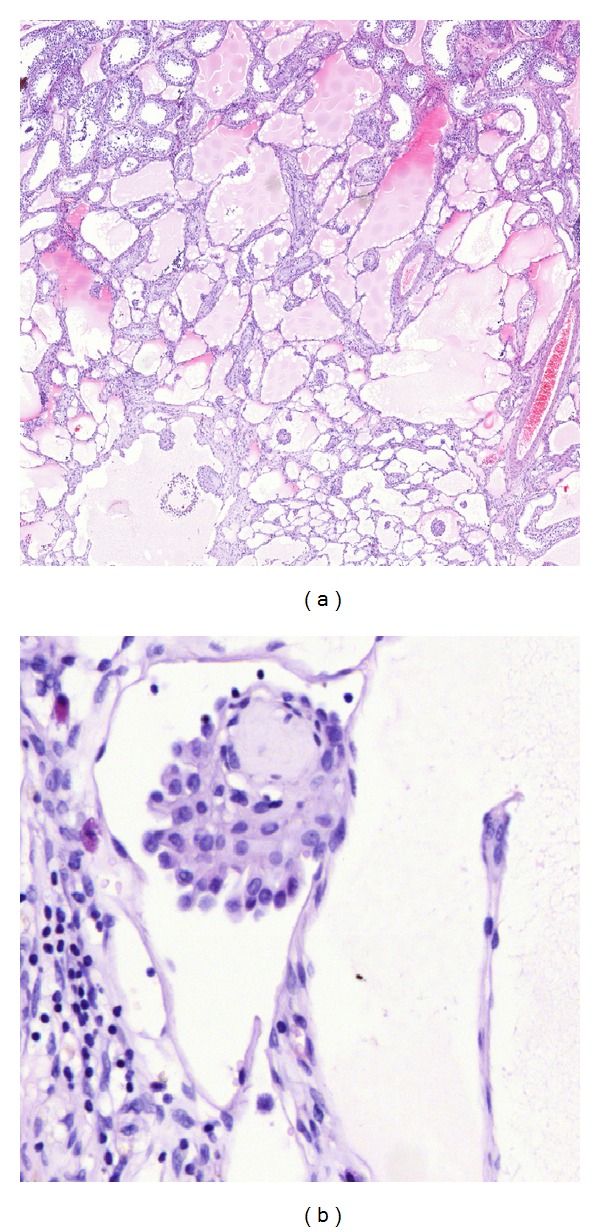
Histomorphology. (a) HE stain 2.5x; (b) HE stain 40x. Images show tumor in relation to adjacent testicular tissue: cavernous vessel structures with prominent intraluminal papillae composed of plump rounded cells with eosinophilic cytoplasm.

Microscopic examination showed a lesion consisting of numerous thin-walled ectatic vascular spaces. Many of these contained proteinaceous material and, hence, closely resembled cavernous lymphangiomas, except for the fact that there were prominent intraluminal papillae composed of plump rounded cells with a limited amount of eosinophilic cytoplasm (Figure 1).

**Figure 2:**
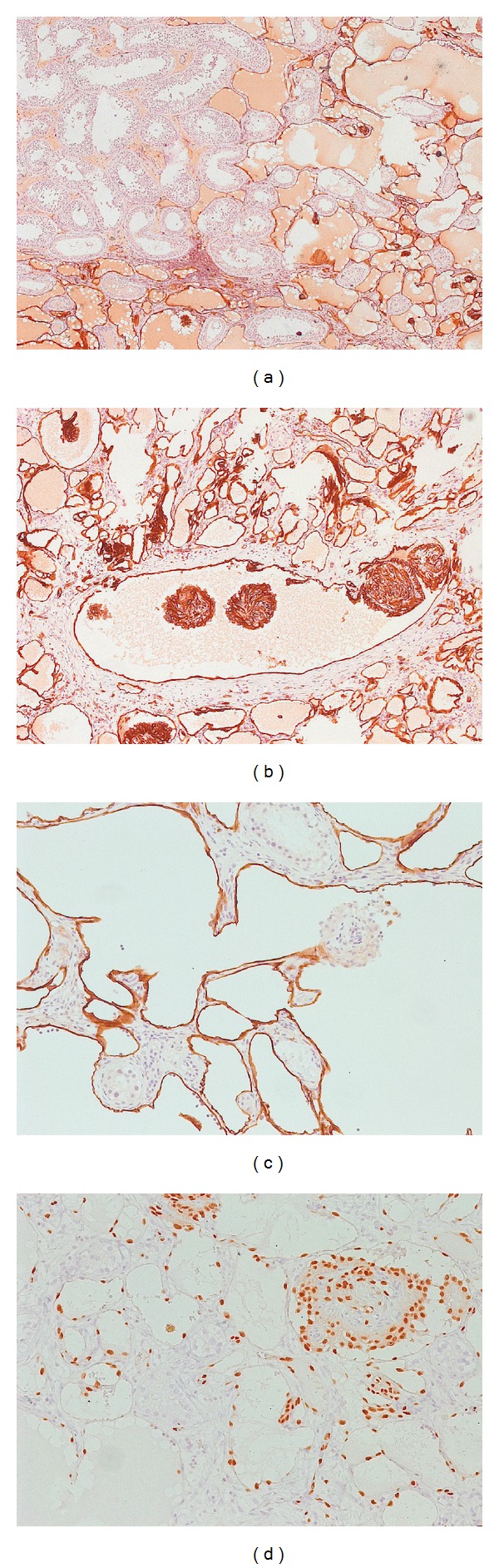
Immunohistochemistry. (a) CD-31 2.5x; (b) CD-31 20x; (c) D2-40 20x; (d) ERG 40x. Images show strong nuclear positivity for ERG in the vessel lining cells, as well as the papillary structures.

Immunohistochemical staining revealed strong nuclear positivity for ERG and CD31 in the cells lining the spaces, as well as the papillary tufts. Staining for D2-40 only showed positivity for the vascular channels, suggesting a lymphatic origin of the vessel structure. The papillaries did not show any positive staining results for D2-40 (Figure 2). 

## 3. Discussion

In 1969, Dabska was the first to describe endovascular papillary angioendothelioma in skin and subcutaneous tissues in 6 cases of children [1]. The tumor was classified as being a low-grade angiosarcoma characterized by papillary endovascular proliferations of atypical endothelial cells and anastomosing vascular channels within the dermis. In 1999, Fanburg-Smith et al. were the first to suggest that the term Dabska tumor should be replaced by a more precise name: papillary intralymphatic angioendothelioma (PILA) [6]. The intention was to discriminate it from a closely related tumor, the retiform hemangioendothelioma.

Since 1969, approximately 30 cases have been described in the literature. The age of the patients ranged from few months to 83 years, and there was no sex predilection [6]. Besides skin and subcutaneous tissue, the tumor has been described in other deeper locations like the spleen [5], soft tissues [7, 8], bone [4], and tongue [3]. The clinical appearance is variable. However, in general, it presents as a slow-growing, usually intradermal nodule that grows up to 2-3 cm in diameter. Symptoms of pain can be present. Microscopically, the cuboidal or hobnail endothelial cells lining the vascular structures are characterized by a high nuclear cytoplasmic ratio and an apically placed nucleus that produces a surface bulge, accounting for the term “hobnail” or “matchstick” [9]. Individual endothelial cells range from cuboidal to tall and cylindrical with vacuolated cytoplasm and hyperchromatic eccentric nuclei on their luminal border. The endothelial cells show only minimal cytologic atypia, and mitotic activity can be scant or absent. Many intraluminal lymphocytes may also be evident, often attached to the endothelial cells. Immunohistochemically, the tumor cells are reported to be positive for Von-Willebrand factor, CD31, CD34, and vascular endothelial growth factor receptor-3 (VEGFR-3) [2, 9]. In the present case, strong nuclear positivity for ERG immunostaining was observed in the vascular space forming tumor cells, as well as the papillary structure forming cells. ERG, an ETS family transcription factor, is known to be expressed in endothelial cells, and oncogenic ERG gene fusions occur in subsets of prostatic carcinoma, acute myeloid leukemia, and Ewing sarcoma. There are new data which suggest it to be a new promising and highly specific marker for vascular endothelial neoplasms [10]. Our findings support this hypothesis.

In our case as well as one other case of the literature, the tumor was located in the testis. Prognostically, these tumors are low-grade lesions with a capacity to metastasize to regional lymph nodes [6]. Three of the original 6 cases were locally aggressive, with tumor invasion into deeper structures, including bone, musculature, fascia, and/or tendons. One of Dabska's original 6 Dabska tumor patients ultimately died of widespread pulmonary metastasis [1]. Therefore, Dabska tumor though believed to have a favorable prognosis can be locally invasive with a potential to metastasize. In the present case, there was no evidence of any nodal involvement or distant metastasis.
